# The Electromagnetic Noise Level Influence on the Laser Micro-Perforation Process Specific to Automotive Components

**DOI:** 10.3390/ma17164131

**Published:** 2024-08-21

**Authors:** Alexandru-Nicolae Rusu, Dorin-Ion Dumitrascu, Adela-Eliza Dumitrascu

**Affiliations:** 1 Department of Manufacturing Engineering, Transilvania University of Brasov, 5 Mihai Viteazul, 500036 Brasov, Romania; alexandru.rusu@unitbv.ro (A.-N.R.); dumitrascu_a@unitbv.ro (A.-E.D.); 2 Department of Automotive and Transport Engineering, Transilvania University of Brasov, 1 Politehnicii, 500036 Brasov, Romania

**Keywords:** laser micro-perforation process, synthetic leather, electromagnetic noise analysis, pull test force, design of experiments (DOE), airbag components

## Abstract

This article focuses on the influence of generated electromagnetic noise (energy) during the micro-perforation process. This study aims to investigate the critical parameters and effects of using laser technology in the processing of textile materials for airbags. Different levels of electromagnetic noise and material thicknesses were investigated to ensure the quality of manufactured parts and the best component performance. A factorial analysis (DOE) was developed to evaluate the influence of electromagnetic noise levels over pull test results and its effect on the micro-perforation process. The overall inferential analysis concludes a significant influence of the noise levels on micro-perforation processing. The detailed analysis suggests that 1.2 V is an optimal level of electromagnetic noise where the material maintains its mechanical properties in a more predictable and consistent manner. Additionally, the factorial design provides significant evidence for an interaction and main effects’ influences of analyzed factors. The obtained results in this study have demonstrated that monitoring and controlling the noise level have beneficial effects over the laser processing. This ensures that the safety aspect of the produced parts is entirely upheld and protected. Also, this research contributes to improving the manufacturing process and ensures that high-quality products are obtained, being suitable for use in sensitive applications such as automotive airbags.

## 1. Introduction

Laser technology has transformed numerous industrial applications, notably in the material processing field, where its precision and efficiency are unmatched [1,2,3,4]. One critical application is in the manufacturing of textile components for airbags. The quality of these components is paramount, as they play a crucial role in ensuring the safety and performance of the final product. The micro-perforation process, specifically, must adhere to strict standards to prevent any compromise in the structural integrity of the airbag materials.

Previous research has focused on optimizing laser parameters for various applications without deeply exploring the effects of electromagnetic noise on the quality and uniformity of laser-perforated holes, especially in critical safety components like airbags. Noise in the context of laser processing refers to unwanted energy variations that can affect the stability and precision of the laser beam. Such variations can lead to inconsistencies in perforation size, shape, and edge quality, all of which are critical parameters for maintaining the functionality and safety of airbags.

Earlier studies have extensively explored the optimization of laser parameters to enhance processing efficiency and material quality for micro-hole perforation. For instance, Guo et al. [5,6] presented the fabrication of surface micro-nanostructures on metals using femtosecond (fs) laser irradiation, leading to significant advancements in creating highly absorptive surfaces. While these studies have provided substantial insights into the structural transformations induced by laser processing, the specific impact of laser noise on the quality and uniformity of the resulting structures has not been thoroughly investigated.

Various researchers have since endeavored to enhance the antireflective characteristics of fs laser-formed surface micro-nanostructures to minimize metal surface reflectance [7,8]. For instance, Iyengar et al. [9] applied fs lasers to produce conical microstructures on titanium surfaces, achieving low surface reflectance values of approximately 3% over a broad spectral range (0.4–1.6 µm) and angular range (0–60°), with the lowest reflectance recorded at about 1.8% for specific wavelengths.

The fabrication speeds of conventional fs lasers make the process time-intensive for producing black metals by focusing fs laser beams directly on sample surfaces. To enhance processing efficiency, Paivasaari et al. [10] proposed a four-beam interferometric fs laser ablation technique, which facilitated the creation of hole-array structures on stainless steel and copper surfaces. Although nearly total absorption was achieved for stainless steel across the 200–2300 nm spectrum, copper samples exhibited a gradual increase in reflectance to approximately 50% at 800 nm. Additionally, nanosecond lasers have been utilized to blacken copper, achieving consistent absorption rates above 97% in the 250–750 nm range through the formation of highly organized periodic microstructures. However, a linear rise in reflectance up to 30% was noted between 750 nm and 2500 nm.

Research efforts have also investigated the antireflective properties of nanoscale structures formed by an fs laser on metal surfaces. Due to their dimensions being comparable to visible light wavelengths, nanoscale structures often exhibit selective optical responses. Dusser et al. [11] created oriented nanostructures on metal surfaces, which induce colorful surface effects and have been leveraged for generating specific color patterns.

Challenges persist in achieving effective light harvesting and minimizing surface reflection across a broad spectrum without wavelength dependence, critical for various applications. This necessitates continuous advancements in antireflection strategies, including conventional quarter-wavelength films, multilayered film stacks for destructive interference [12], direct moth-eye mimics, nanowire/porous-based dielectric structures for the gradient refractive index [13,14], single-scale metallic micro- or nano-features, multiscale hierarchical structures for light trapping [15], meticulously designed and fabricated metamaterials, and their patterned and dimension-varied counterparts for inducing resonance in ε and μ individually [16]. Ongoing research is also exploring novel light-harvesting approaches [17,18,19,20,21,22].

A significant breakthrough in antireflection research has been the development of coatings comprising vertically aligned carbon nanotubes, achieving ultra-low reflectance (<1%) across an ultra-broadband spectrum (from UV to far-infrared) [23,24]. Furthermore, advancements in ultra-broadband light harvesting from UV to THz ranges have been achieved by fabricating nanotip arrays on silicon surfaces, highlighting the potential for enhancing antireflection properties on metal surfaces.

From the perspective of micro-nanostructure fabrication, considerable challenges remain in achieving the designable, predictable, controllable, and scalable fabrication of surface micro-nanostructures across various materials. Addressing these challenges remains a continuous pursuit among researchers aiming to develop general strategies and techniques for fabricating such structures.

## 2. Objectives and Scope of This Study

This study focuses on the influence of generated noise (energy) during the laser micro-perforation process of synthetic leather airbag components. Noise in the context of laser processing refers to the unwanted energy variations that can affect the stability and precision of the laser beam. Such variations can lead to inconsistencies in perforation size, shape, and edge quality, all of which are critical parameters for maintaining the functionality and safety of airbags. By closely monitoring and controlling noise levels, the aim is to optimize the production process, ensuring that the resulting airbag components meet stringent quality and safety standards. This approach not only enhances the overall efficiency of the manufacturing process but also contributes to the development of high-quality products suitable for critical applications in the automotive industry.

In this context, this paper details the experimental analysis, including the equipment and methodologies used, and presents a comprehensive assessment of the results. To assess the effects of electromagnetic noise levels on pull test force results, a factorial analysis was applied to underline the influences of the main effects and interactions of examined factors. The study findings will underscore the importance of controlling laser noise to maintain the integrity and performance of airbag components, ultimately contributing to advancements in the field of laser material processing.

The research from this paper presents a niche in the field of the impact of electromagnetic noise on the quality and consistency of laser micro-perforations in textile materials for airbags. Previous studies have largely concentrated on optimizing laser parameters without exploring deeply into how external factors like electromagnetic noise influence the process. By filling this gap, the research provides valuable insights into how controlling electromagnetic noise can improve the manufacturing process and enhance the safety and reliability of airbag components.

## 3. Materials and Methods

### 3.1. Materials and Equipment

The material subjected to testing is synthetic leather made from polyvinyl chloride (PVC) [25], with a nominal thickness of 0.8 mm. It is laminated with a spacer material having a nominal thickness of 2.99 mm and a specified tolerance of ±0.3 mm. The combined material must meet strict strength and flexibility criteria to ensure airbag functionality.

The synthetic leather parts used in this study were selected to represent common materials utilized in the automotive component manufacturing industry for airbag production. The sample size consisted of 400 parts for each analyzed parameter: the laser noise, material thickness, and pull test results. For the factorial analysis, 1200 samples were considered.

The used equipment:

- ZwickRoell Z100 Testing Machine (Ulm, Germany) features a maximum force capacity of 100 kN. It provides a testing speed range from 0.0005 to 1000 mm/min with an accuracy of ±0.1% of the set value. It ensures force measurement accuracy within ±0.5% of the measured value, up to 1/1000 of the maximum load cell capacity. The machine has a test stroke of 1100 mm without accessories and operates on a power supply of 230 V, 50/60 Hz.
- The INSTRON 5967 Testing Machine (Norwood, MA, USA), with a maximum force capacity of 30 kN, offers a testing speed range from 0.001 to 3000 mm/min with an accuracy of ±0.1% of the set value. It ensures force measurement accuracy within ±0.5% of the measured value, up to 1/1000 of the maximum load cell capacity. The machine has a test stroke of 1130 mm without accessories; operates on a power supply of 100–240 V, 50/60 Hz; and utilizes Bluehill Universal software 4.5.

The ZwickRoell Z100 testing machine is used for evaluating the mechanical properties of laser-processed materials. Its features, such as high force capacity and measurement precision, make it ideal for testing textile materials, ensuring compliance with technical specifications.

The INSTRON 5967 testing machine is used for smaller-scale testing and applications requiring lower forces. With similar accuracy and flexible power supply, it complements the capabilities provided by the ZwickRoell Z100.

### 3.2. Evaluation Methodology of the Quality of Laser Micro-Perforations in Airbag Zones’ Components

The quality of laser micro-perforations’ process applied to materials used for airbags is evaluated based on several critical parameters. The size and uniformity of the perforations must be consistent across the entire material surface, as variations can compromise the functionality of the airbag. The ideal shape of the perforations is circular, and any deformation may indicate issues with laser parameter settings or beam quality.

The quality of the perforation edges is also essential; they should be smooth and well defined to prevent material degradation during airbag deployment. The depth of the perforations must be precisely controlled to ensure optimal performance, thereby avoiding any risk of structural failure.

The material surrounding the perforations must maintain its structural integrity without showing signs of thermal or mechanical degradation. It is important that the perforation process minimizes residue generation, as the presence of residues can affect both the esthetics and functionality of the airbag.

The efficiency and speed of the micro-perforation process are important parameters for industrial production. An optimal balance between speed and quality is necessary to maintain efficient and economical production. To achieve high-quality perforations, it is crucial to optimize and precisely control laser parameters such as power, frequency, scanning speed, noise (energy in the electromagnetic field), and beam focus. The use of appropriate calibration equipment and regular maintenance significantly contributes to maintaining optimal performance.

For the analysis of the quality of laser micro-perforations in airbag zones, three test samples, each containing 400 pieces, were examined. These samples underwent a rigorous set of tests to evaluate the influence of the previously mentioned parameters: power and noise (energy in the electromagnetic field). The results of these tests provided essential data to ensure compliance with the safety and performance standards specific to the automotive industry.

### 3.3. Laser Processing

**Figure 1:**
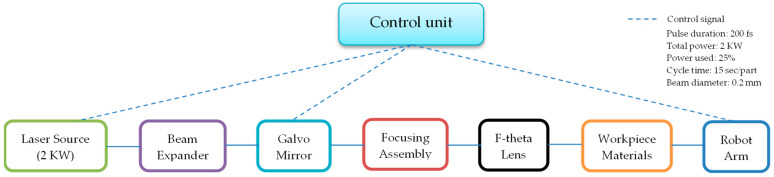
Schematic diagram of experimental setup with robot integration.

**Figure 2:**
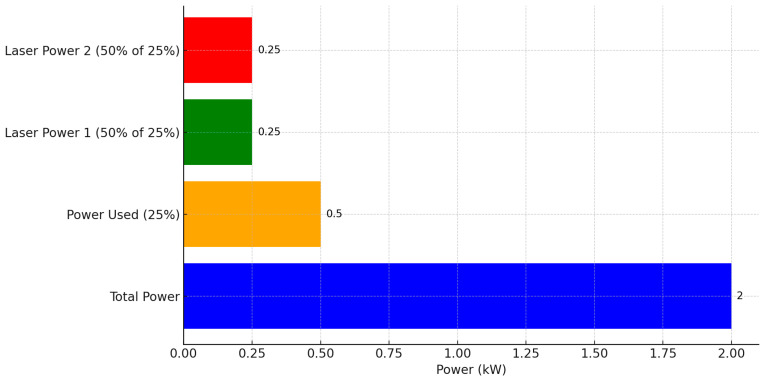
Laser power settings and distribution.

The laser used in this experiment has a total power of 2 kilowatts (kW), representing 100% of the device nominal power. The experimental settings include using 25% of the total power, while the two laser powers are set at 50% used from this percent. Additionally, within this 0.5 kW setting, the powers P1 and P2 are adjusted to 50% of 0.5 kW, equating to 0.25 kW each. The measurement of the laser power is conducted in discrete increments of 100 watts, up to the full 2 kW capacity, to ensure a comprehensive evaluation of the laser efficiency across its operational range. These settings are crucial for evaluating the performance and efficiency of the material perforation process (Figure 1 and Figure 2). The pulse duration is 200 fs (femtosecond). This is unusual for CO_2_ lasers, which traditionally have much longer pulse durations (in the order of nanoseconds). However, there are advanced technologies where different types of lasers are combined to achieve very short pulse durations, such as the Ti series pulsed CO_2_ lasers.

With a production cycle of 15 s per part, it is crucial that the quality of the part in the visible area is ensured throughout the vehicles’ lifespan. This means that micro-perforations resulting from the laser processing must not be visible and should not be influenced by factors that could compromise the integrity of the airbag or the esthetic appearance of the components.

**Figure 3:**
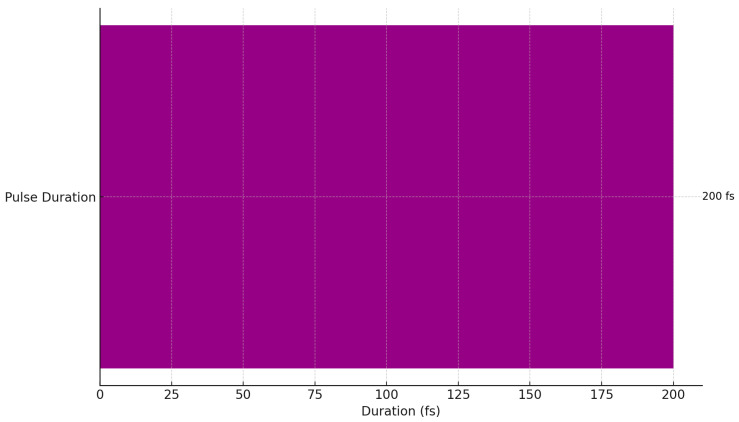
Laser pulse duration.

In conclusion, the Ti series pulsed CO_2_ laser with a pulse duration of 200 fs (Figure 3) is a costly but efficient option for precision applications in airbag component production, ensuring high quality and long-term durability of the manufactured parts.

### 3.4. Optical Configuration

**Figure 4:**
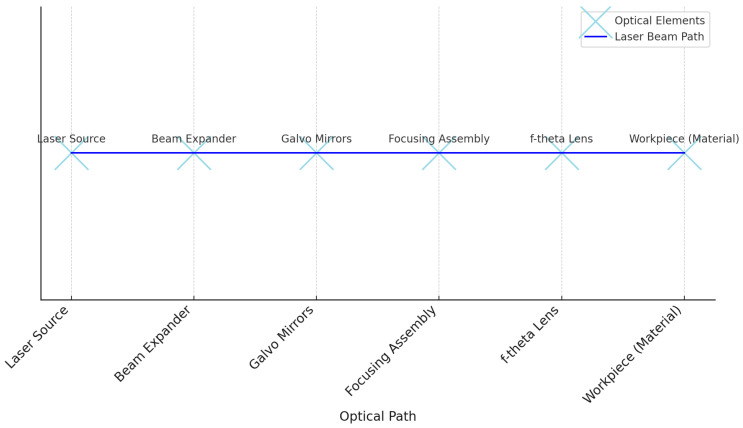
Optical configuration of laser system.

The laser optical system includes f-theta scanning lenses, beam expanders, focusing assemblies, and Galvo mirrors, all designed to operate at the wavelengths of CO_2_ lasers. The beam diameter is 0.2 mm, ensuring precise focusing. The focal length of the optical system depends on the specific type of lenses and mirrors used. For f-theta scanning lenses and Galvo mirrors, the focal length can be adjusted according to application requirements. The beam size is controlled by the optical system and can be adjusted to obtain a small focal point, essential for precise processing of textile materials (Figure 4).

The material is placed precisely at the focal plane of the optical system. This ensures optimal focusing of the laser beam and precise processing of the material.

### 3.5. Collection and Statistical Analysis

The experiments were conducted with meticulous attention, taking into account the imposed conditions and using advanced instrumentation and equipment.

The statistical analyses of the experimental data were performed using the Minitab v17 software (Minitab LLC., State College, PA, USA). Considering a 95% confidence interval (CI) and a significance level of α = 0.05, the normal distribution of the experimental data was qualitatively and quantitatively validated by using the normal Anderson–Darling test (AD) [26,27]. A factorial design (DOE) [28,29] was applied based on the process particularities by choosing the main control factors that affected the micro-perforation characteristics. To evaluate the influence of the electromagnetic noise levels, the interaction effects and main effects of factors were studied.

## 4. Influence of Electromagnetic Noise (EMI) on Laser Processing

During the experiment, electromagnetic noise (EMI) was monitored and controlled to understand its impact on the quality of laser perforations. Electromagnetic noise can be generated from various sources in the surrounding environment and can affect the stability and precision of the laser beam.

To evaluate the influence of EMI, pull tests were performed on the perforated materials under different electromagnetic noise conditions.

### 4.1. Statistical Analysis of Experimental Data

**Figure 5:**
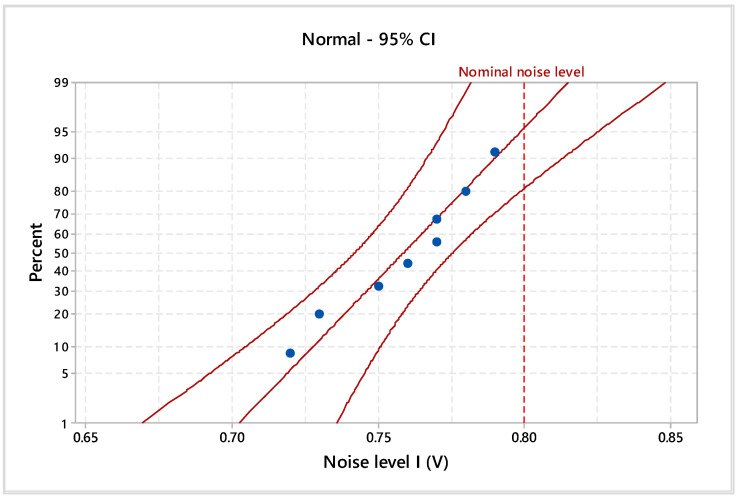
Probability plot of noise level I for 0.8 V.

**Figure 6:**
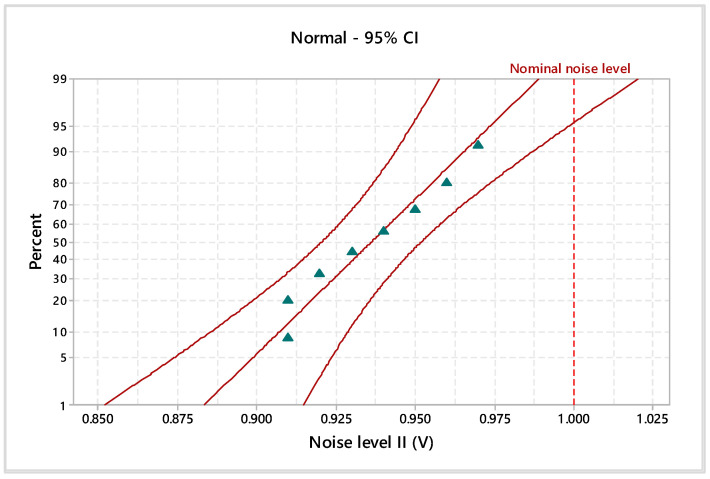
Probability plot of noise level II for 1 V.

**Figure 7:**
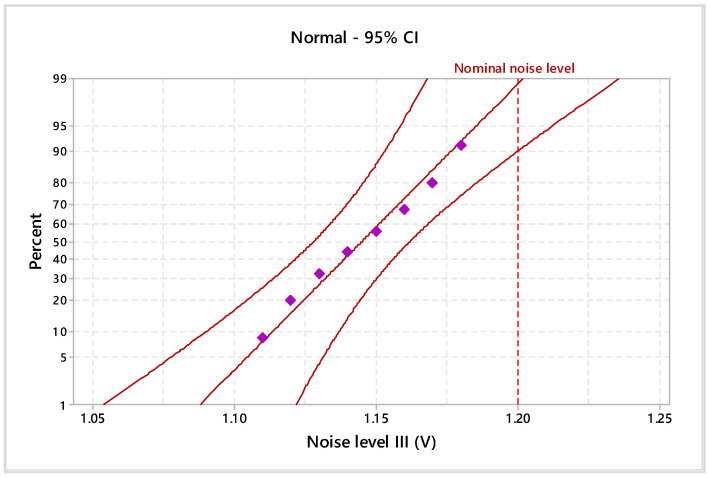
Probability plot of noise level III for 1.2 V.

**Figure 8:**
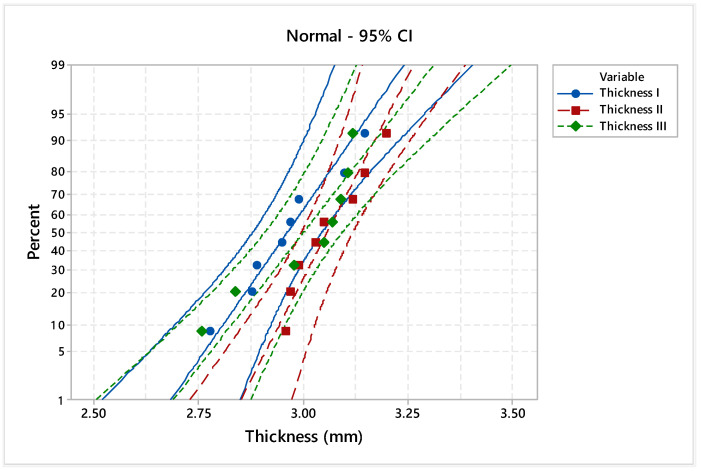
Probability plot of analyzed material thicknesses.

Considering the 95% confidence interval (CI), the electromagnetic noise levels and materials’ thicknesses were statistically analyzed. In particular, the Anderson–Darling normality test was applied to validate the data distribution. Additionally, the homogeneity of the experimental data was tested by assessing the goodness of fit with probability plots (Figure 5, Figure 6, Figure 7 and Figure 8), indicating the nominal electromagnetic noise level.

The goodness-of-fit normality test results are presented in Table 1. The comparative analysis of the probability plots shows that all points are within the lower and upper confidence boundaries, respectively, and the *p*-value is over the specific significance level of 0.05. Based on the estimated Anderson–Darling statistics (AD) and correlation coefficient, it can be mentioned that the analyzed experimental data are homogeneous and it follows the normal distribution.

The descriptive statistics of analyzed electromagnetic noise levels and material thicknesses are synthetically presented in Table 2 and Table 3.

The estimated mean of electromagnetic noise level I is 0.759 V (95% confidence intervals of 0.738 V and 0.778 V), the standard deviation is 0.024 V (95% confidence intervals of 0.015 V and 0.049 V), and the median is 0.765 V (95% confidence intervals of 0.729 V and 0.780 V). With a significance level of α = 0.05, the estimated parameters do not exceed the imposed nominal level of 0.8 V. Based on descriptive statistics results for electromagnetic noise levels II and III, it indicates falling within the specified limits for 1 V and 1.2 V.

In the case of the material thicknesses, the means are between 2.964 mm and 3.059 mm, and the standard deviations are between 0.089 mm and 0.134 mm with medians around 3 mm. Although the differences between the main estimated statistical parameters are not significant, each piece is analyzed individually, and the material thickness influences the regime of the laser process.

### 4.2. Analysis of the Main Factors in the Laser Micro-Perforation Process

**Figure 9:**
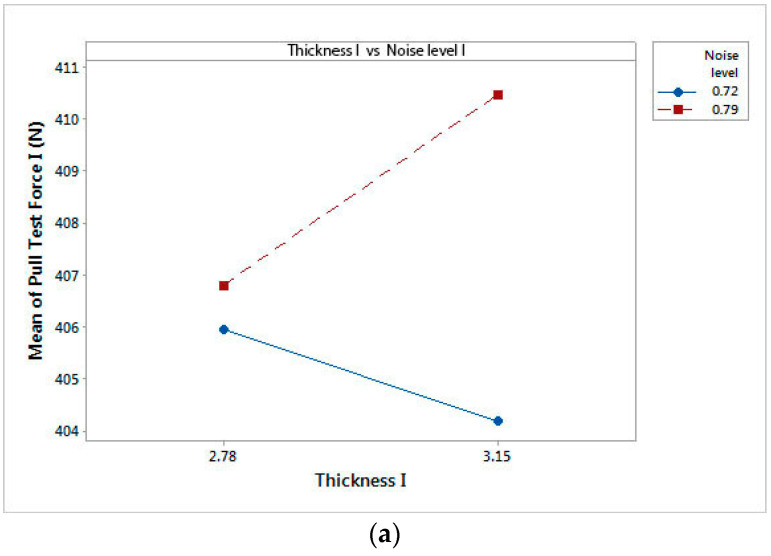

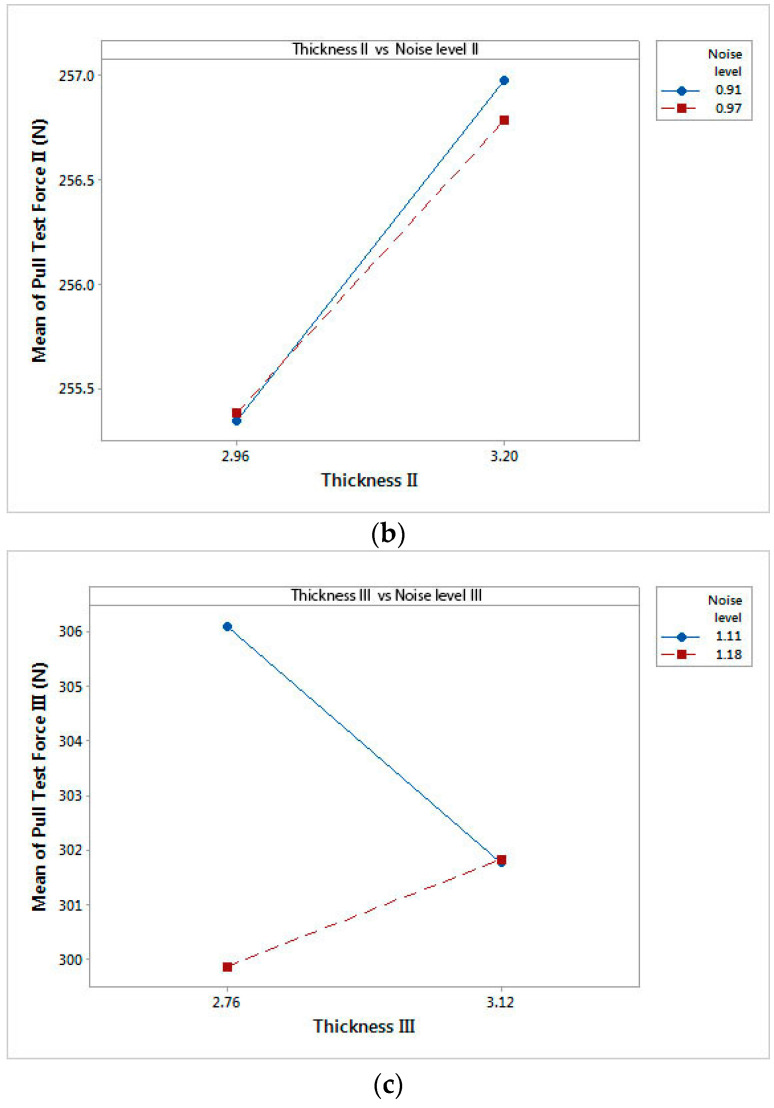
Interaction plot of analyzed factors: (**a**) interaction effects of pull test force I; (**b**) interaction effects of pull test force II; (**c**) interaction effects of pull test force III.

**Figure 10:**
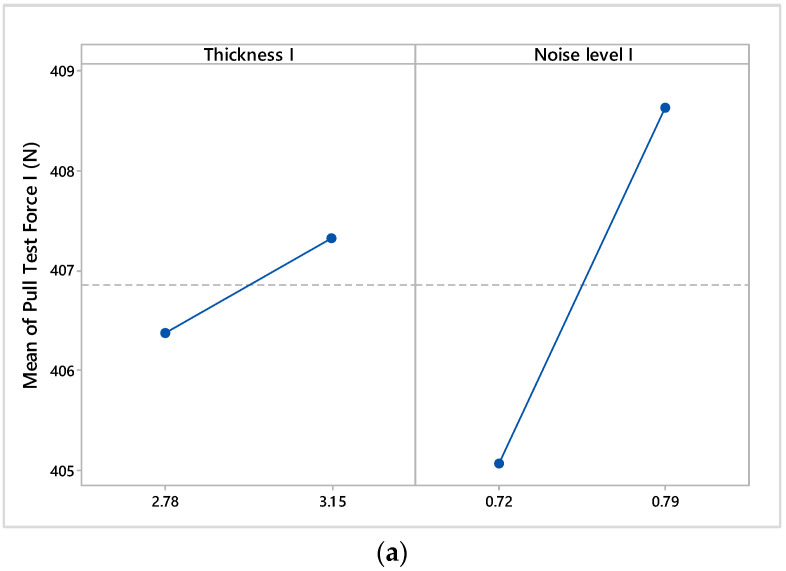

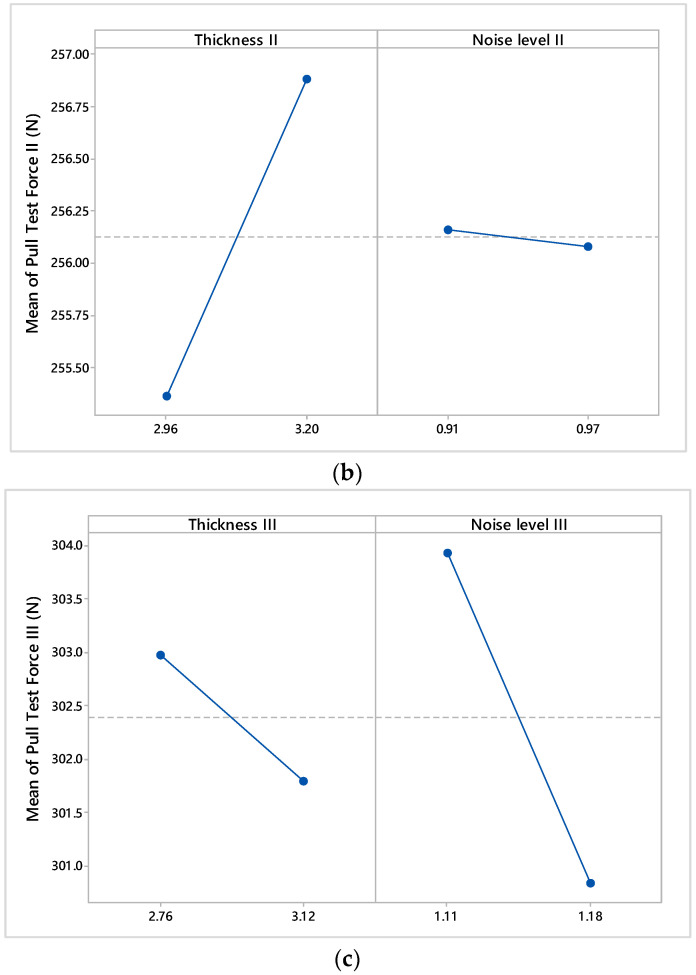
Main effects plot of analyzed factors: (**a**) main effects for pull test force I; (**b**) main effects for pull test force II; (**c**) main effects for pull test force III.

The influence of electromagnetic noise levels and material thicknesses over pull test results is based on the factorial design of experiments (DOE). The goal of this study is to examine these factors to determine which ones have the greatest influence. Because it was assumed that three-way and four-way interactions are negligible, a resolution IV factorial design was adopted. We decided to generate a 16-run fractional factorial design. The interaction effects and main effects for factorial design results are presented in Figure 9 and Figure 10.

The interaction plots show that there is a high degree of interaction for pull test force I and pull test force III. The electromagnetic noise level with the highest values has significant influence on pull test force results (Figure 9a,c). Specific to pull test force II depicted in Figure 9b, the plot indicates no interaction.

The pull tests’ forces are adjusted according to the thickness of the material and its texture, the target value of the pull test being 303 N. From Figure 9a, it follows that for a value of noise level I of 0.79 V, the force for the pull test is 410 N, this value not falling within the agreed tolerance. From Figure 9b, it can be seen that for 0.97 V, the maximum value recorded in the pull test is 257 N. This result does not reflect the fulfillment of the imposed requirements. Analyzing Figure 9c, the pull test force value is 302 N at a noise level of 1.18 V, a result that indicates the supply of compliant products, but also the stability of the laser micro-perforation process.

The main effects’ plots indicate that the electromagnetic noise levels and material thicknesses influence the results of pull tests (Figure 10). Specifically, the noise levels compare to a nominal noise level of 0.8 V that has a negative influence on the results of the pull test, while, for a nominal noise level of 1.2 V, it has a positive influence.

## 5. Results and Discussion

### 5.1. Evaluation of Perforation Quality in the Presence of Electromagnetic Noise

The pull tests conducted on the perforated materials were analyzed to determine the impact of electromagnetic noise on perforation quality. The tests revealed that, in the presence of electromagnetic noise, there were significant variations in the size and shape of the holes, as well as the presence of irregular edges.

The structural examination of the perforated material using the Gemini 500 Zeiss electron microscope (Zeiss, Jena, Germany) revealed the presence of defects such as excessive burns and deformations of holes’ edges in areas exposed to strong electromagnetic fields. These defects can compromise the integrity and functionality of the airbag, highlighting the necessity of EMI control in the laser process.

### 5.2. Analysis of Tensile Test Results

**Figure 11:**
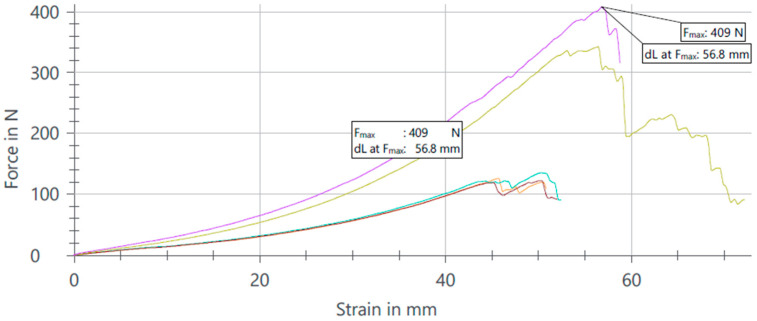
Analysis results after pull test with NOK values and noise set to 0.8 V.

**Figure 12:**
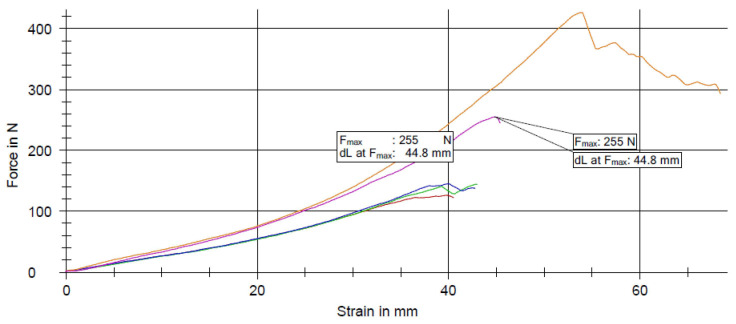
Analysis results after pull test with NOK values and noise set to 1 V.

**Figure 13:**
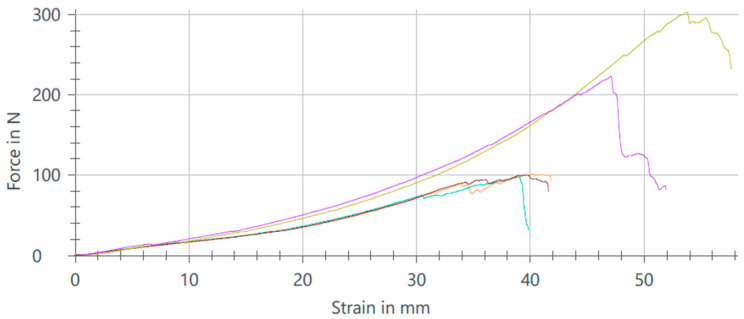
Analysis results after pull test with OK values and noise set to 1.2 V.

The results of the tensile tests reveal distinct performance characteristics between samples categorized as NOK (Not OK) and those categorized as OK (Figure 11, Figure 12 and Figure 13). Specifically, samples classified as NOK exhibited an average tensile strength of 409 N at a noise level of 0.8 V, and an average of 255 N at a nominal electromagnetic noise level of 1 V. In contrast, samples categorized as OK demonstrated an average tensile strength of 303 N when exposed to a noise level of 1.2 V. These findings underscore the sensitivity of tensile strength to variations in applied noise levels, highlighting the need for a precise control and optimization of experimental parameters in material testing protocols.

These results indicate that the tensile values are affected by the level of electromagnetic noise. At a noise level of 1.2 V, the tensile values are more consistent and closer to the optimal values, demonstrating that this level is favorable for achieving the desired quality of perforations.

### 5.3. Impact of Electromagnetic Noise on the Material

The 1.2 V noise level appears to favor greater consistency and stable quality of perforations compared to lower or higher noise levels (Figure 13). This noise level helps maintain a balanced mechanical performance and reduces variability in the size and shape of perforations.

Under 1.2 V noise conditions, defects and deformations are minimized, ensuring better structural integrity of the perforated material.

### 5.4. Relationship between Noise and Mechanical Performance

The results suggest that an electromagnetic noise level of 1.2 V offers an optimal balance between mechanical performance and consistency of results. While lower noise levels (0.8 V) provide higher tensile values, they are more variable and less predictable. At 1.2 V, the perforated material shows superior stability, which is crucial for critical applications such as airbags.

The perforated material was subjected to strength tests in the presence of electromagnetic noise, using the ZwickRoell Z100 and INSTRON 5967 testing machines. The results showed a decrease in mechanical strength in areas affected by EMI, emphasizing the importance of minimizing electromagnetic interference to maintain the final product quality.

### 5.5. Process Optimization

Laser processing of textile components for airbags has a significant impact on their quality and performance. Laser technology allows high precision and consistency in material perforation, essential for ensuring the functionality and safety of airbags. However, the influence of electromagnetic noise can compromise these advantages, affecting the stability and accuracy of the laser process.

Using lasers in processing textile materials offers numerous advantages, such as the ability to achieve precise and uniform perforations and the efficiency of the process. However, the presence of EMI can introduce defects and variabilities that reduce the final product quality.

To improve the laser processing process and minimize the influence of electromagnetic noise, the following main recommendations can be mentioned:

- Electromagnetic Shielding: Continue using electromagnetic shielding equipment to maintain the optimal noise level at 1.2 V.
- Experimental Setup Isolation: Maintain the adequate isolation of the experimental setup to prevent unexpected variations in electromagnetic noise.
- Power Supply Stabilization: Ensure a stable power supply to consistently maintain the optimal electromagnetic noise level at 1.2 V.

During the laser perforation of the airbag’s protective material, electrostatic energy is generated, which can impact the process. However, the primary objective of this procedure is to ensure the precise and efficient perforation of the material without compromising the functionality or safety of the airbag.

To prevent any adverse effects and ensure the reliability of the airbags in the event of an impact, specific measures are implemented. The electrostatic energy produced by the laser device can potentially affect nearby materials or electronic components under certain conditions. Therefore, measures are taken to minimize electrostatic interference and to safeguard the integrity of both the airbag system and the associated electronic components.

To enhance the micro-perforation process and control the level of electrostatic noise, adjustable tolerances are utilized. These tolerances can be configured through the laser’s software, allowing parameters to be adjusted based on the material used for micro-perforation. This ensures that the perforation is carried out effectively without compromising the performance and safety of the airbag.

In this regard, the studies on influences of electromagnetic noise show that these factors must be detected, controlled, and mitigated.

## 6. Conclusions

Laser processing of textile components for airbags is a complex and crucial field for vehicle safety. By deeply understanding process parameters and the influence of external factors such as electromagnetic noise, superior results can be achieved that comply with the industry stringent specifications. Continuing research and continuous improvement of the technologies and methodologies used will ensure the development of high-quality products and maximum safety for end-users.

In conjunction with the design of experiments’ analysis, the differences among the analyzed factors were examined. The three noise levels appear to affect the pull test results compared to a target value of pull test force. The interaction effect is present because the different levels of the analyzed electromagnetic noise factors affect the response differently. Additionally, the plots of influence of electromagnetic noise levels compare to material thickness over pull test force results; it shows that there is a main effect present. Based on the analysis of factor effects, it can be mentioned that the noise levels have significant influence over the micro-perforation laser process.

The unacceptable results demonstrate significant variation in tensile values under the influence of electromagnetic noise. At a noise level of 0.8 V, the average tensile value is 407 N, suggesting better mechanical performance but with reduced consistency. At a noise level of 1 V, the average tensile value drops to 256 N, indicating a significant deterioration in the quality of the perforated material.

The acceptable results show that at a noise level of 1.2 V, the average tensile value is 303 N. Although the absolute value is lower than that at 0.8 V, the consistency and stability of the results are superior.

A detailed analysis of the tensile test results under the influence of electromagnetic noise indicates that a noise level of 1.2 V is optimal for achieving the desired quality of perforations in textile materials for airbags. This noise level provides a balance between mechanical performance and consistency of results, minimizing defects and ensuring the necessary structural integrity for vehicle safety. Controlling and maintaining this optimal electromagnetic noise level will significantly contribute to improving the quality and reliability of the final products.

## Figures and Tables

**Table 1:** Anderson–Darling normality test.

Characteristic	AD	Correlation Coefficient	*p*-Value
Noise level I (V)	0.247	0.979	0.649
Noise level II (V)	0.225	0.980	0.731
Noise level III (V)	0.134	0.996	0.961
Material thickness I (mm)	0.198	0.985	0.826
Material thickness II (mm)	0.287	0.972	0.524
Material thickness III (mm)	0.615	0.919	0.407

**Table 2:** Descriptive statistics of analyzed noise level.

Statistical Parameter	Noise Level I (V)	Noise Level II (V)	Noise Level III (V)
Mean	0.759	0.936	1.145
Minimum	0.720	0.910	1.110
Maximum	0.790	0.970	1.180
Median	0.765	0.935	1.145
StDev	0.024	0.023	0.024
Q1	0.735	0.913	1.123
Q3	0.778	0.958	1.168
Skewness	−0.54	0.23	0.010
Kurtosis	−0.744	−1.412	−1.200

**Table 3:** Descriptive statistics of analyzed material thicknesses.

Statistical Parameter	Material Thickness I (mm)	Material Thickness II (mm)	Material Thickness III (mm)
Mean	2.964	3.059	3.003
Minimum	2.780	2.960	2.760
Maximum	3.150	3.200	3.120
Median	2.960	3.040	3.060
StDev	0.120	0.089	0.134
Q1	2.883	2.975	2.875
Q3	3.073	3.143	3.105
Skewness	0.201	0.487	−1.159
Kurtosis	−0.287	−1.264	−0.035

## Data Availability

The data are contained within the article.
